# Modeling the Spread of Crimean Congo Hemorrhagic Fever with Tick Co-feeding

**DOI:** 10.1007/s11538-026-01777-2

**Published:** 2026-10-28

**Authors:** Jessica L. Kingsley, Amber A. Young, Stella A. Atim, Stephen Balinandi, Simbarashe Chitanga, Faraimunashe Chirove, Hannah E. Correia, F. Stephen Dobson, Christina J. Edholm, Paul D. Kasiaja, Praise-God Madueme, Kelly Joëlle Gatore Sinigirira, Michael L. Washington, Holly Gaff, Suzanne Lenhart

**Affiliations:** 1 Department of Mathematics, University of Tennessee, Knoxville, TN 37996 USA; 2 Department of Mathematics, Spelman College 350 Spelman Ln SW, Atlanta, GA 30314 USA; 3 National Animal Disease Diagnostics and Epidemiology Center, Entebbe, Uganda; 4 The Viral Pathogens Theme: MRC/UVRI & LSHTM, Uganda Research Unit, Entebbe, Uganda; 5 Uganda Virus Research Institute, Entebbe, Uganda; 6 Department of Preclinical Studies, School of Veterinary Medicine, University of Namibia, Windhoek, Namibia; 7 Department of Mathematics and Applied Mathematics, University of Johannesburg, Johannesburg, South Africa; 8 Department of Biology, University of North Dakota, Grand Forks, ND 58202 USA; 9 Department of Biological Sciences, Auburn University, Auburn, AL 36849 USA; 10 Department of Mathematics, Scripps College, Claremont, CA 91711 USA; 11 National Livestock Resources Research Institute (NaLIRRI), National Agricultural Research Organization, Kampala, Uganda; 12 Center for Climate Physics, Institute for Basic Science, Busan, South Korea; 13 Department of Mathematical Sciences, University of Burundi, Bujumbura, Burundi; 14 Michael Washington Consultancy, Pretoria, South Africa; 15 Department of Biological Sciences, Old Dominion University, Norfolk, VA 23529 USA

**Keywords:** Ordinary differential equations, Tick-borne illnesses, Co-feeding, Crimean Congo hemorrhagic fever, * Hyalomma* & *Rhipicephalus*

## Abstract

Crimean Congo hemorrhagic fever (CCHF) is a viral disease in animals and humans. Ticks transmit the virus that causes this disease to susceptible hosts by feeding on them, and ticks can acquire the virus by feeding on an infected host. The tick species most commonly associated with CCHF feed on different hosts at different stages in their life cycle: larvae and nymphs feed on small mammals, while adults feed on large mammals, like humans and cattle. An additional transmission pathway, co-feeding, occurs when infected and susceptible ticks feed in close proximity on the same host, allowing viral transmission between ticks even if the host remains uninfected. In this work, we developed the first system of ordinary differential equations capturing CCHF transmission that includes co-feeding dynamics, seasonal variation in tick populations, and all three feeding life stages of ticks with their subsequent hosts. We adapted the model to Uganda’s Cattle Corridor, a region with frequent human–cattle interactions and reported CCHF activity. Simulations explored both baseline and immigration event scenarios, capturing realistic ecological and epidemiological dynamics. By varying the co-feeding transmission, we found notable effects on the hosts and ticks, including a shift in timing of seasonal infection peaks and changes to the number of human infections from ticks or cattle. The inclusion of additional tick life stages demonstrates how co-feeding strengthens viral circulation within ticks and increases host infectivity. These results highlight the importance of including ecological complexity in models of CCHF transmission and to illustrate the effects of co-feeding.

## Introduction

Crimean Congo hemorrhagic fever (CCHF) is a viral tick-borne disease affecting countries across Africa, Asia, and Europe, making it the most widespread viral hemorrhagic fever (Aslam et al. 2016; CDC 2025). CCHF is designated as a priority pathogen by the U.S. National Institute for Allergy and Infectious Diseases (National Institute of Allergy and Infectious Diseases (NIAID) 2025). For humans, symptoms usually begin a few days after infection, with sudden high fever, strong headaches, muscle aches, and tiredness. Other symptoms often include dizziness and gastrointestinal problems. In severe cases, the illness can progress to bleeding under the skin and low platelet counts (World Health Organization 2023; Ergönül 2006; CDC 2025). To diagnose CCHF, experts rely on a variety of laboratory tests. In the early days of illness, the virus can be detected in blood samples using Polymerase Chain Reaction (PCR), with subsequent identification of viral genetic material (Sas et al. 2018). Another test, antigen Enzyme-Linked Immunosorbent Assay (ELISA), can detect viral proteins (Burt et al. 1998). After about five days, the host body begins to produce antibodies against the virus, and antibodies can be measured using blood tests for immunoglobulin M (IgM); recent infection) and immunoglobulin G (IgG); longer-term response) (Dowall et al. 2012; Yadav et al. 2022). Antibodies for CCHF can remain present for up to 5 years (Frank et al. 2024). Most human cases of CCHF do not result in severe symptoms; however, fatality rates among those with severe symptoms can be up to 15% (World Health Organization 2023). Case fatality rates for all reported infections range from 3% to 40% (Kizito et al. 2018; WHO 2025).

Crimean Congo hemorrhagic fever is spread to humans through a tick bite or by interaction with bodily fluids or blood of an infected host (Frank et al. 2024). Ticks of the genus *Hyalomma* and *Rhipicephalus* have been identified as vectors and their range expansions have contributed to the spread of CCHF (Gordon et al. 1993; Lule et al. 2022). Ixodid ticks have four stages to their life cycle: eggs, larvae, nymphs, and adults. For the species we consider, the latter three stages take blood meals from various hosts and feed on different hosts at different stages in their life cycle: larvae and nymphs feed on small mammals and birds, and adults feed on large mammals (Aslam et al. 2016). Another pathway of transmission for some tick-borne pathogens is transmission between the vectors, called co-feeding transmission. Co-feeding transmission occurs when an infected tick and an uninfected tick feed in close proximity on the same host, and the infected tick transmits the pathogen to the uninfected tick, while the host remains uninfected (Belli et al. 2017). Co-feeding transmission can occur via a variety of biological mechanisms, one being that the virus is transmitted through the saliva of the ticks on the surface of the host (Gargili et al. 2017). Co-feeding’s impact on disease transmission has been studied for a few tick-borne illnesses, including Lyme disease and CCHF (Belli et al. 2017; Gordon et al. 1993; Voordouw 2015).

Mathematical models of tick-borne illnesses have been helpful tools to capture transmission dynamics and inform control strategies. Some models of tick-borne illnesses with ordinary differential equations have been proposed that incorporate co-feeding transmission (Zhang et al. 2021; Tosato et al. 2021). There are also ordinary differential equation models that are specific to CCHF that include co-feeding transmission and consider humans, adult ticks, and livestock (Bhowmick et al. 2022; Switkes et al. 2016). Switkes et al. modeled CCHF, conducted a thorough $R_0$ analysis of the disease spread, and discussed critical parameter combinations that contribute to the rate of spread (Switkes et al. 2016). Bhowmick et al. published another ordinary differential equations model of CCHF that accounted for tick co-feeding, with compartments for humans, livestock, and adult ticks. They also conducted an $R_0$ analysis, as well as a sensitivity analysis and numerical simulations using the model (Bhowmick et al. 2022). They indicated the need for further models with seasonality.

The goal of our work is to develop a realistic ODE model of CCHF that incorporates tick co-feeding, three life stages of ticks, as well as human, cattle, and small mammal hosts. Additionally, we use our model to explore potential promoters of disease spread like seasonality and immigration events tied to human cultural events. We investigate the effects of co-feeding by simulating our model with various proportions of co-feeding transmission. Our model of CCHF is also applied to Uganda’s Cattle Corridor by including seasonality and resulting oscillatory tick populations, as well as reasonable tick class sizes. Numerical simulations of both baseline and immigration event scenarios are conducted, and the immigration event scenarios reflect the Cattle Corridor’s social practices.

Because our simulations explore how different ecological and behavioral factors influence human infection outcomes, it is important to clarify how these outcomes relate to the epidemiological measures commonly reported in the literature. In epidemiological studies, prevalence refers to the percentage of current infections detected by PCR or antigen tests, while seroprevalence refers to percentage of past exposure identified through antibody testing, which can remain positive long after the infectious period has passed (Dowall et al. 2012; Yadav et al. 2022). For CCHF, non-severe cases are often misdiagnosed (Raabe 2020) and seroprevalence testing is often conducted only after a confirmed human case (Atim et al. 2023). Consequently, published prevalence and seroprevalence estimates may substantially underrepresent the true extent of infections among all hosts. These limitations motivate our use of modeled seroprevalence and prevalence to examine potential promoters of human risk in settings where empirical data remain sparse.

In the Model section, we present the structure of our model and its parameters. The Results with Scenarios section provides numerical simulations and their interpretations. Finally, the Discussion and Conclusions section highlights the key findings of this study and proposes directions for future research.

## Model

**Fig. 1:**
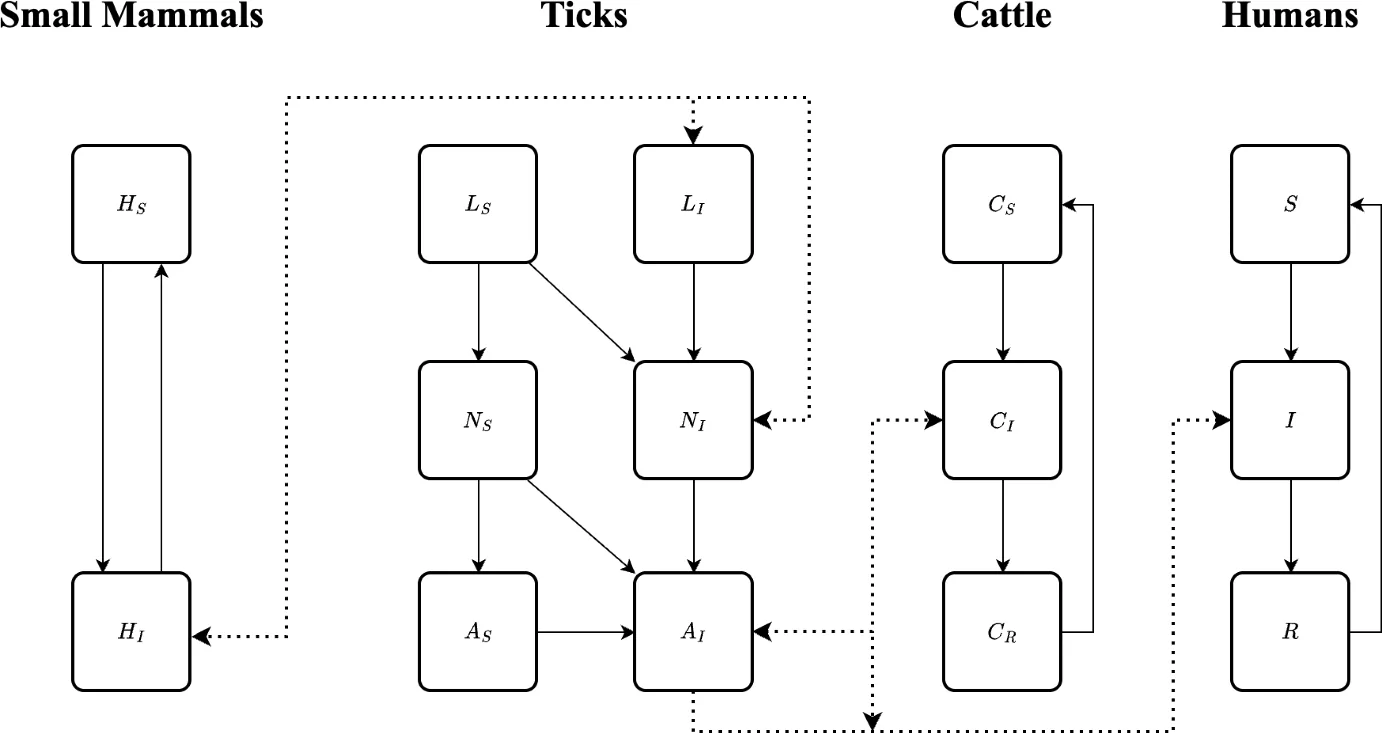
Class transitions of model (1)–(2). Small mammal hosts can transition back and forth from susceptible and infected, $H_S$ and $H_I$. Human and cattle hosts can transition to and from susceptible, infection, and recovered, *S*, *I*, *R* and $C_S, C_I, C_R$ respectively. Ticks progress forward in their life stages and can remain susceptible or become infected in each progression. They cannot become susceptible once they are infected. Larvae are denoted by $L_S$ and $L_I$, nymphs are $N_S$ and $N_I$, and adults are $A_S$ and $A_I$. Dashed arrows denote classes contributing to infection among ticks and hosts

Our model is a system of fourteen ordinary differential equations which includes larval, nymphal, and adult ticks, as well as small mammals, cattle, and human hosts. Co-feeding dynamics are captured in six of the tick differential equations. State variables are summarized in Table 1, and parameters are summarized in Tables 2 and 3. The host state variables are: susceptible and infected small mammal hosts ($H_S, H_I$); susceptible, infected, and recovered cattle ($C_S, C_I, C_R$); and susceptible, infected, and recovered humans (*S*, *I*, *R*). The following eight differential equations describe our host dynamics. Transitions among the state variables are illustrated in Fig. 1.

1 $$\begin{aligned} \begin{aligned} \frac{dH_S}{dt}&= \Lambda _{H} - \beta _L L_I H_{S} - \beta _{N} N_{I} H_{S} + \gamma _{H} H_I-\mu _H H_S\\ \frac{dH_I}{dt}&= \beta _L L_I H_{S} + \beta _{N} N_{I} H_{S} - \gamma _{H} H_I-\mu _H H_I\\ \frac{dC_S}{dt}&= \Lambda _{C} - \beta _{A} A_{I} C_{S} + \rho _{C} C_R-\mu _{C} C_S\\ \frac{dC_I}{dt}&= \beta _{A} A_{I}C_{S} - \gamma _{C} C_I -\mu _C C_I\\ \frac{dC_R}{dt}&= \gamma _{C} C_I - \rho _{C} C_R-\mu _C C_R\\ \frac{dS}{dt}&= - \beta _1 S A_I - \beta _2 S \frac{C_I}{c_2 +C_I} +\rho R\\ \frac{dI}{dt}&= \beta _1 S A_I +\beta _2 S \frac{C_I}{c_2 +C_I} -\gamma I -\epsilon I\\ \frac{dR}{dt}&= \gamma I -\rho R \end{aligned} \end{aligned}$$

The following six differential equations describe the tick dynamics, with susceptible and infected for each of the three life stages: larvae (*L*), nymphs (*N*), and adults (*A*).

2 $$\begin{aligned} \begin{aligned} \frac{dL_S}{dt}&= \Lambda _L\bigl (A_S + (1-\theta )A_I\bigr ) - \bigl (\alpha _H H + \mu _L\bigr ) L_S\\ \frac{dL_I}{dt}&= \Lambda _L \theta A_I - \bigl (\alpha _H H + \mu _L\bigr ) L_I \\ \frac{dN_S}{dt}&= \alpha _H s_N L_S \Bigg [ (1-\epsilon _L)H_I + H_S \frac{L_S + N_S + (1-\eta _{LL})L_I + (1-\eta _{NL})N_I}{L+N} \Bigg ] \\&\quad - \bigl (\alpha _H H + \mu _N\bigr ) N_S \\ \frac{dN_I}{dt}&= \alpha _H s_N \Bigg [ L_S \Big ( \epsilon _L H_I + H_S \frac{\eta _{LL}L_I + \eta _{NL}N_I}{L+N} \Big ) + L_I H \Bigg ] - \bigl (\alpha _H H + \mu _N\bigr ) N_I \\ \frac{dA_S}{dt}&= \alpha _H s_A N_S \Bigg [ (1-\epsilon _N) H_I + H_S \frac{L_S + N_S + (1-\eta _{LN})L_I + (1-\eta _{NN})N_I}{L+N} \Bigg ] \\&- \Bigg ( \alpha _C \epsilon _A C_I + \alpha _C \eta _{AA} C_S \frac{A_I}{A} + \mu _A \Bigg ) A_S \\ \frac{dA_I}{dt}&= \alpha _H s_A \Bigg [ N_S \Big ( \epsilon _N H_I + H_S \frac{\eta _{NN}N_I + \eta _{LN}L_I}{L+N} \Big ) + N_I H \Bigg ] \\&+ \Bigg ( \alpha _C \epsilon _A C_I + \alpha _C \eta _{AA} C_S \frac{A_I}{A} \Bigg ) A_S - \mu _A A_I \end{aligned} \end{aligned}$$

Small mammal hosts enter the susceptible population at rate $\Lambda _H$ and the infected hosts recover at rate $\gamma _H$. Susceptible small mammals become infected by interactions with infected larvae $L_I$ and infected nymphs $N_I$ at rates $\beta _L$ and $\beta _N$ respectively, corresponding to the terms $\beta _LL_IH_S$ and $\beta _NN_IH_S$. Small hosts can only exit the population by natural death at rate $\mu _H$.

Similarly, the susceptible cattle population increases at a constant influx rate $\Lambda _C$. When cattle recover, they become temporarily immune to the virus, $\gamma _C C_I$. When their immunity wanes, they enter the susceptible class at rate $\rho _CC_R$ that represents the waning of immunity. Susceptible cattle become infected with rate $\beta _AA_IC_S$ according to mass action interaction with infected adult ticks $A_I$. Cattle may also die with natural mortality rate $\mu _C$. Cattle enter the infected class at a rate of $\beta _A A_I C_S$ and leave when they recover at a rate of $\gamma _CC_I$. Then the cattle in the recovered class develop immunity and leave at a rate $\rho _CC_R$ to become susceptible.

Susceptible humans can become infected according to interaction with infected adult ticks at the rate $\beta _1SA_I$. Their interaction with cattle is represented with a Holling Type 2 function (Holling 1959)

$$\begin{aligned} \beta _2 S \frac{C_I}{c_2 +C_I} \end{aligned}$$

with half saturation constant $c_2$, and infection rate $\beta _2$ to represent the transmission of the disease from cattle to humans via blood and bodily fluids. The susceptible class increases when recovered humans lose their temporary immunity at rate ρR. Humans may die from the disease with rate ϵI, and recover to become temporarily immune with rate γI. They enter the recovered class with rate γI and leave at a rate of ρR. We did not consider either birth or natural death rates, as the human population we consider reflects a relatively stable adult subgroup engaged in routine cattle-related activities. Over the five-year simulation period, demographic turnover is unlikely to substantially alter the size or composition of this group.

All tick classes include associated natural mortality rates, larvae $\mu _L$, nymph $\mu _N$, and adults $\mu _A$. The susceptible larval class increases from the laying and successful molting of eggs from susceptible adults $\Lambda _LA_S$ and from cases without vertical transmission $\Lambda _L(1-\theta )A_I$, where θ is the proportion of successful vertical transmission. Susceptible larvae leave the class by taking a blood meal from any small host with biting rate $\alpha _H HL_S$, where $H=H_S+H_I$. The infected larval class increases from an infected adult successfully transmitting the disease to an egg that survives $\Lambda _{L \theta A_I}$. Infected larvae leave their class when by taking a blood meal from any small host, $\alpha _H HL_I$.

Larval ticks become nymphs through a blood meal on a host, which larvae may not survive, so we multiply by a survival rate $s_N$. For susceptible larval ticks, they become susceptible nymphs when susceptible larvae feed on infected hosts and direct infection does not occur, i.e., $(1-\epsilon _L)L_S H_I$, or when susceptible larvae feed on susceptible hosts and co-feeding infection does not occur. Previously infected larvae remain infected and progress to infected nymphs when they feed on any small host, $\alpha L_I H$.

**Fig. 2:**
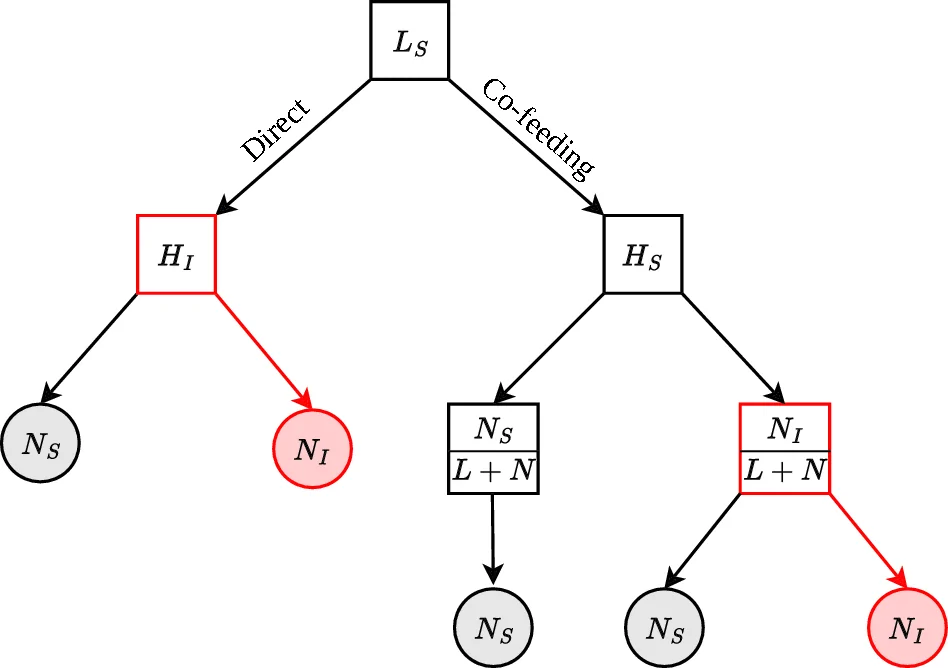
Possible progressions from the susceptible larval class to the infected and susceptible nymphal classes. Pathways to susceptible nymphs are depicted with black arrows and gray nodes, while pathways to infected nymphs are depicted with red arrows and red nodes. The leftmost branch shows direct transmission, with the other branches showing co-feeding transmission with a nymph (Color figure online)

Figure 2 illustrates the potential pathways a susceptible larval tick can take to enter into the susceptible and infected nymphal classes, including co-feeding with a nymph. For the direct branch, susceptible larvae can become infected through feeding on an infected small host with rate $\epsilon _L L_S H_I$, or, as mentioned above, infection doesn’t occur and the susceptible larva become susceptible nymphs. For the co-feeding branch, we consider the ratios of susceptible and infected nymphs to the total larval and nymphal populations, where $L=L_S+L_I$ and $N=N_S+N_I.$ If a susceptible nymph is encountered, the susceptible tick feeds and progress to become a susceptible nymph with term

$$\begin{aligned} \displaystyle \alpha L_S H_S \frac{N_S}{L+N}. \end{aligned}$$

Otherwise, if an infected nymph is encountered, we consider if the co-feeding infection is successful, letting $\eta _{ij}$ be the proportion of co-feeding transmission between a susceptible tick *j* and an infected tick *i* that occurs and is successful. Therefore, if an infected nymph is encountered and co-feeding transmission is not successful, the susceptible larva progresses to the susceptible nymphal class with term

$$\begin{aligned} \displaystyle \alpha L_S H_S \frac{(1-\eta _{NL})N_I}{L+N}. \end{aligned}$$

Meanwhile, if co-feeding transmission is successful, the susceptible larva becomes an infected nymph by

$$\begin{aligned} \displaystyle \alpha L_S H_S \frac{\eta _{LN}N_I}{L+N}. \end{aligned}$$

If instead susceptible larvae encounter nymphs during co-feeding, the larvae progress to susceptible nymphs through

$$\begin{aligned} \alpha L_S \frac{L_S}{L+N} \ \text {or} \ \displaystyle \alpha L_S H_S \frac{(1-\eta _{LL})L_I}{L+N}. \end{aligned}$$

Otherwise, the susceptible larvae progress to an infected nymph through co-feeding infection success with

$$\begin{aligned} \displaystyle \alpha L_S H_S \frac{\eta _{LL}L_I}{L+N}. \end{aligned}$$

For previously infected larvae, they remain infected and progress to infected nymphs when they feed on any small host, $\alpha L_I H$.

Nymphs progressing to susceptible or infected adults is similar to the progression from larvae to nymphs. We include a feeding rate associated with the required small mammal blood meal of $$\alpha _H$$, and a survival rate associated with nymphs feeding $$s_A$$, multiplied on all nymphal terms. Susceptible nymphs may become susceptible adults by feeding on an infected small host and infection does not occur, $$\alpha (1-\epsilon _N) N_S H_I$$ or by unsuccessful co-feeding with either larvae or nymphs on a susceptible small host. Meanwhile, infected nymphs remain infected and progress to infected adults when they take a blood meal on any small host, $$ N_I H$$. The nymphal co-feeding terms in the susceptible and infected adult equations are analogous to the larval co-feeding terms in the nymphal equations: susceptible nymphs remain susceptible when they encounter other susceptible ticks,

$$\begin{aligned} N_S H_S \frac{L_S}{L+N} \ \text {or} \ N_S H_S \frac{N_S}{L+N}. \end{aligned}$$

Susceptible nymphs also remain susceptible when they encounter infected ticks on a susceptible small host and co-feeding transmission is unsuccessful:

$$\begin{aligned} N_S H_S \frac{(1-\eta _{LN})L_I}{L+N} \ \text {or} \ N_S H_S \frac{(1-\eta _{NN})N_I}{L+N}. \end{aligned}$$

If direct or co-feeding transmission is successful, then the nymphs progress to infected adults with similar terms and parameters $\epsilon _N$,$\eta _{LN}$,and $\eta _{NN}.$

Adult ticks are the only tick stage that can remain within their class after taking a blood meal. We model adults feeding on cattle with $\alpha _C$ exclusively because the cattle are the only large host that can transmit the virus. A susceptible adult can become infected when it feeds on infected cattle, $\epsilon _A A_S C_I$ or when it co-feeds with an infected adult on susceptible cattle. The adult co-feeding term includes a ratio of infected adult ticks to the total adult population, $\frac{A_I}{A}$, with $A=A_S+A_I$. The term corresponding to successful co-feeding transmission between adult ticks is

$$\begin{aligned} \eta _{AA} C_SA_S\frac{A_I}{A}. \end{aligned}$$

The adult death rate $\mu _A$ includes natural death and the potential of death associated with feeding on both cattle and humans.

**Table 1:** State variables and descriptions

Symbol	Description
$H_S$	Susceptible small hosts
$H_I$	Infected small hosts
*H*	Total small host population
$C_S$	Susceptible cattle
$C_I$	Infected cattle
$C_R$	Recovered cattle
*S*	Susceptible humans
*I*	Infected humans
*R*	Recovered humans
$L_S$	Susceptible larvae
$L_I$	Infected larvae
*L*	Total larval population
$N_S$	Susceptible nymphs
$N_I$	Infected nymphs
*N*	Total nymphal population
$A_S$	Susceptible adults
$A_I$	Infected adults
*A*	Total adult population

**Table 2:** Host parameters, baseline values, and units

Symbol	Description	Value	Units
$\Lambda _H$	Recruitment rate of small mammals (sm)	100	${(\text {sm} \cdot \textrm{day})}^{-1}$
$\beta _L$	Transmission rate from infected larvae to small mammals	$8\times 10^{-5}$	${(\textrm{ticks}\cdot \textrm{day})}^{-1}$
$\beta _N$	Transmission rate from infected nymphs to small mammals	$8\times 10^{-5}$	${(\textrm{ticks}\cdot \textrm{day})}^{-1}$
$\mu _H$	Natural mortality rate of small mammals	0.01	$\textrm{day}^{-1}$
$\gamma _H$	Recovery rate of small mammals^a^	0.083	$\textrm{day}^{-1}$
$\Lambda _C$	Recruitment rate of susceptible cattle	4.445	cattle/day
$\beta _A$	Transmission rate from infected adult ticks to cattle	$4.2\times 10^{-5}$	${(\textrm{ticks}\cdot \textrm{day})}^{-1}$
$\rho _C$	Waning immunity rate of cattle^a^	0.00833	$\textrm{day}^{-1}$
$\mu _C$	Natural mortality rate of cattle	$5.5\times 10^{-4}$	$\textrm{day}^{-1}$
$\gamma _C$	Recovery rate of infected cattle^a^	0.05	$\textrm{day}^{-1}$
$\beta _1$	Tick-to-human transmission rate	$1.125\times 10^{-6}$	${(\textrm{ticks}\cdot \textrm{day})}^{-1}$
$\beta _2$	Cattle-to-human transmission rate	$5\times 10^{-4}$	$\textrm{day}^{-1}$
$c_2$	Saturation constant for cattle-to-human	500	cattle
ρ	Waning immunity rate for humans^a^	0.00833	$\textrm{day}^{-1}$
γ	Human recovery rate^a^	0.05	$\textrm{day}^{-1}$
ϵ	Disease-induced mortality rate in humans	0.001	$\textrm{day}^{-1}$

^a^Consistent with reported antibody dynamics for CCHF (Frank et al. 2024)

**Table 3:** Tick parameters, baseline values, and units

Symbol	Description	Value		Units
θ	Proportion of larvae infected via vertical transmission	0.15		unitless
$s_N$	Proportion of larvae transitioning to nymphs	0.25		unitless
$\epsilon _L$	Proportion of direct larval infection	0.05		unitless
$\eta _{ij}$	Proportion of co-feeding transmission^a^	0.05		unitless
$s_A$	Proportion of nymphs transitioning to adults	0.15		unitless
$\epsilon _N$	Proportion of direct nymphal infection	0.05		unitless
$\epsilon _A$	Proportion of direct adult transmission by cattle	0.05		unitless
		Dry	Wet	
$\Lambda _L$	Egg production rate per adult female tick^b^	0.35	0.4	$\hbox {day}^{-1}$
$\alpha _H$	Tick feeding rate on small mammals (sm) ^b^	$4\times 10^{-7}$	$2.5\times 10^{-6}$	$(\textrm{sm} \cdot \textrm{day})^{-1}$
$\alpha _C$	Tick feeding rate on cattle (c)^b^	$4\times 10^{-7}$	$2.5\times 10^{-6}$	$(\textrm{c} \cdot \textrm{day})^{-1}$
$\mu _L$	Larval mortality rate^b^	0.01	0.00015	$\hbox {day}^{-1}$
$\mu _N$	Nymphal mortality rate^b^	0.0095	0.0020	$\hbox {day}^{-1}$
$\mu _A$	Adult mortality rate^b^	0.01	0.0027	$\hbox {day}^{-1}$

Except where noted, values are based on rates for *Hyalomma* ticks and CCHF disease reported in Matser et al. (2009)

Egg production rates, feeding rates, and natural mortality rates were varied bi-annually to reflect seasonality (dry, wet) restrictions (Nimusiima et al. 2013; Kabi et al. 2014; Kasaija 2021). ^a^ *i* and *j* index tick life stages (*L*, *N*,  or *A*) for the infecting tick (*i*) and susceptible tick (*j*) in a cofeeding event. ^b^Varied bi-annually to reflect rainfall seasonality (dry, wet) (Nimusiima et al. 2013; Kabi et al. 2014; Kasaija 2021); see Sect. 3 for details

## Results with Scenarios

CCHF infections have been reported in Uganda as early as 1958, and increased surveillance for the disease has been implemented since 2013. Currently, a single confirmed human case of CCHF initiates a national response (Kizito et al. 2018; Balinandi et al. 2021). Most reports of CCHF in Uganda are from the “Cattle Corridor", a broad agricultural zone of the country that stretches from the southwest to the northeast (Zalwango et al. 2024; Mirembe et al. 2021). The Cattle Corridor is home to the highest populations of cattle in Uganda and is characterized by cattle farming (National Agricultural Research Laboratories (NARL) 2025). We consider a population of one thousand humans in a subcounty of the Cattle Corridor and assumed that they interact with cattle. We balance natural births and deaths to keep a cattle population of around 8,000 for the study area. This choice is consistent with national livestock data in Uganda: Uganda’s National Livestock Census 2021 reports about 8.72 million cattle in the Cattle Corridor and explains that the Cattle Corridor is comprised of 559 sub-counties across the districts (Uganda Bureau of Statistics 2024). This corresponds to an average of about 15,600 cattle per sub-county. In our model, however, the human population represents only the subset of individuals within a sub-county who regularly and directly interact with cattle (e.g., herders, cattle keepers, abattoir workers, or others whose routine activities involve close contact with cattle). Because this subgroup constitutes only a fraction of the total sub-county population, we assume an effective local herd of around 8,000 cattle to reflect the portion of cattle most relevant to the contact patterns of this occupationally exposed group. Explicit counts of small mammals, like rodents and shrews, in the Cattle Corridor are not readily available. Therefore, we choose a small mammal population of around 10,000, reflecting a ratio of one human to 100 rodents, which is consistent with East African agricultural landscapes (Ssuuna 2020; Shilereyo et al. 2023).

To estimate the order of magnitude of ticks for our chosen population, we use the mean number of ticks per host published in Matser et al. (2009). We consider the values indicated for *Hyalomma* and *Rhipicephalus* ticks, which have been attributed to the spread of CCHF (Gordon et al. 1993; Lule et al. 2022). For larvae, the number of ticks per host vary from 16–90 between the two tick species (Matser et al. 2009). With 10,000 hosts for the larvae, we expect the population to be on the order of hundreds of thousands. For the nymphs, the average number per host vary from 6.8 to 21, therefore with the same number of hosts as the larvae, we expect the nymphal population to be on the order of tens of thousands. For the adult ticks, the average number per host is 4.5 to 8.5. The total number of hosts for the adult ticks is about 9000, since they feed on humans and cattle. We choose to model a conservative number of adults that can reasonably feed on the cattle and humans in our region and keep the adult tick population on the order of thousands.

For our initial conditions in all simulations we consider no infection in the humans, resulting in our susceptible population being 1,000 with no humans in infected and recovered. For the other host populations, we choose their initial infected populations to be 1% of their initial susceptible population (Yessinou et al. 2025). We take initial susceptible cattle to be 8,000 with initial infected 80 and initial susceptible small mammals to be 10,000 with 100 infecteds. For ticks we choose initial susceptible populations with orders of magnitudes as described above: larvae 100,000, nymphs 15,000, and adults 7,000. Based on reported CCHF values for ticks, the number of initial infecteds for nymphs and adults is 2% of their respective susceptible populations (Belobo et al. 2021). For larvae we set the initial infecteds to 400 to align with the behavior of the infected curves.

All model parameters are presented in Table 2 (host parameters) and Table 3 (tick parameters). Recruitment and natural mortality rates for cattle and small mammals are chosen for this model to reflect the assumptions for livestock and small mammal counts described above. We choose transmission proportions,the saturation constant, and disease induced mortality rates for this model based on field, veterinary, and entomological expertise among the authors. Recovery rates for all hosts reflect an infectious period of about 3 weeks, which is consistent with both IgM and IgG antibodies peaking at around 3 weeks post infection (Frank et al. 2024). Waning immunity rates for humans and cattle reflect about four months of temporary immunity, which is consistent with IgM antibodies being undetectable after about 4 months (Frank et al. 2024).

We take the feeding rates for ticks to be the same and define $\alpha = \alpha _C=\alpha _H$. To further adapt our model to the Cattle Corridor, we incorporate seasonality through cycling specific tick parameters according to seasons with increased rainfall (wet seasons) and decreased rainfall (dry seasons): December, January, February and June, July, August are the dry seasons, and March, April, May and September, October, November are the wet seasons (Nimusiima et al. 2013). Higher tick abundance is expected in wet seasons compared to dry seasons due to increased humidity (Kabi et al. 2014; Kasaija 2021). To capture the cyclic behavior, the feeding rate α, natural death rates $\mu _L, \ \mu _N, \ \mu _A$, and the influx of larvae $\Lambda _L$ are functions of time. A higher feeding and larval influx rate and lower death rates are used in the wet seasons, while lower feeding and influx and higher death rates are used in dry seasons. We start our simulations in the beginning of a dry season, December 1. For each of the parameters α, $\mu _L, \ \mu _N, \ \mu _A$, $\Lambda _L$, we create a yearly vector that is divided by the four wet and dry seasons and input the corresponding seasonal values outlined in Table 3. In a dry season, we use the smaller of the two values for α and $\Lambda _L$ and the larger of the two values for $\mu _L, \ \mu _N, \ \mu _A$. In a wet season, we use the larger of the two values for α and $\Lambda _L$ and the smaller of the two values for $\mu _L, \ \mu _N, \ \mu _A$. We capture these parameters by taking a discrete function with steps for each season change and doing an interpolation to formulate a continuous function. We simulate our model using MATLAB ode45 numerical method, which uses the interpolated functions on its grid. All other tick parameters are based on values outlined for *Hyalomma* ticks and CCHF disease in Matser et al. (2009).

**Fig. 3:**
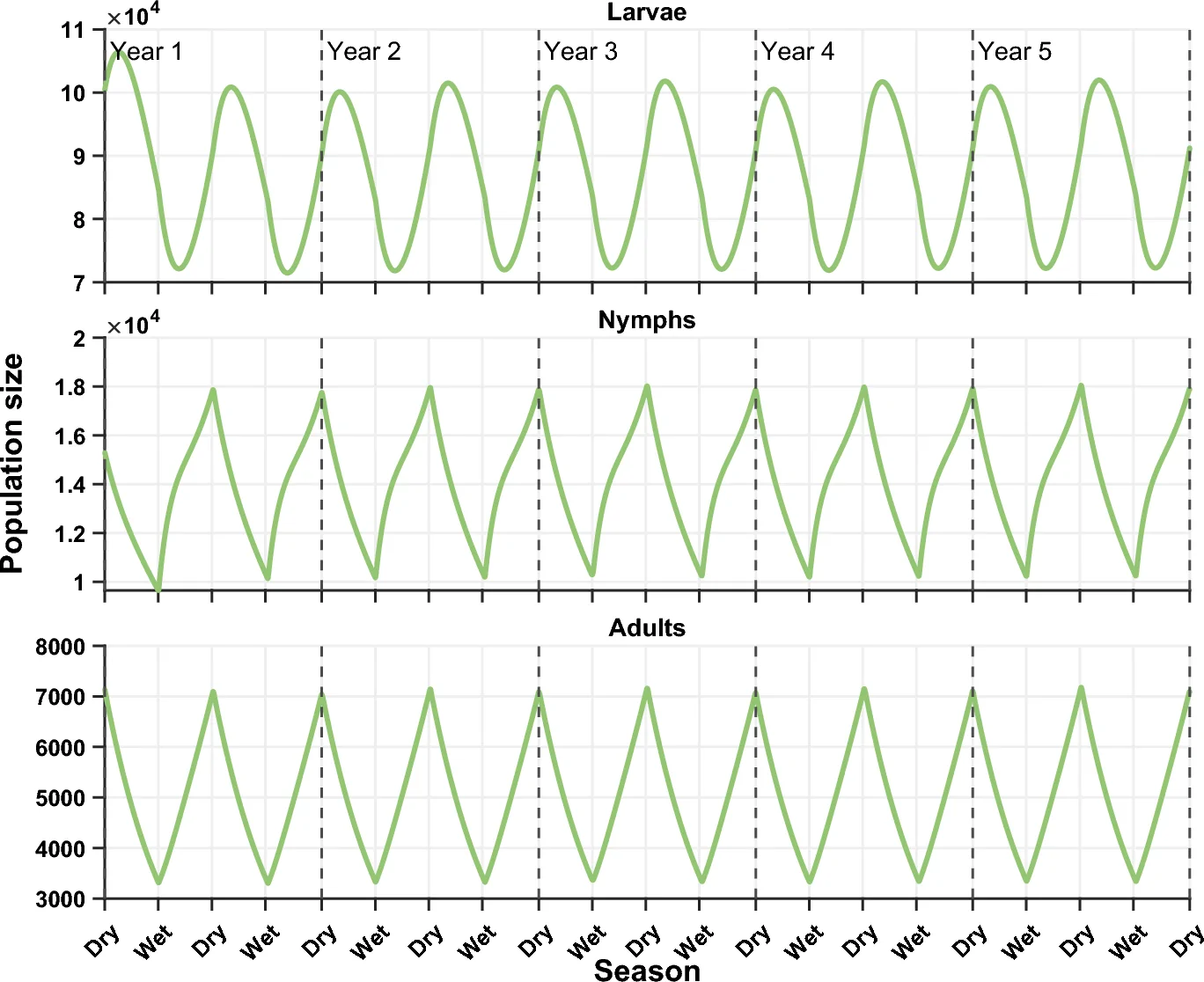
The total population (susceptible and infected) for larval, nymphal, and adult ticks for a simulation ran for 5 years. The horizontal axis displays the rainfall seasonality by highlighting the beginning of each wet and dry season, 2 per year. Dashed vertical lines highlight the transition between years, and years are labeled in the top panel (Color figure online)

The cyclic behavior of the total tick populations (the sum of susceptible and infected ticks) is shown in Fig. 3 for a simulation run for 5 years with the above conditions.

Every simulation includes this cyclic behavior in the tick populations and begins on December 1st, in a dry season. First, we consider the effects of co-feeding on disease dynamics. To study the disease spread, we consider the seroprevalence of CCHF in humans and cattle and the prevalence in small hosts and ticks. We consider seroprevalence in humans as the sum of the infected and recovered classes divided by the total number of humans (and similarly for cattle), while prevalence of a specific group (hosts or ticks) is the infected class divided by the total number of individuals in that group. This choice was made to reflect the majority of available data in the literature. Most of the data collected on CCHF tested for antibodies, which can be present in hosts after their infectious period for up to five years (Frank et al. 2024). Since for ticks and small mammals we have infected and susceptible classes only, we calculate disease prevalence as the number of infected individuals divided by the total number of individuals.

For our simulations, we consider two different possible scenarios: a baseline and immigration events. For the baseline scenario, Sect. 3.1, initially there are only infections in ticks and hosts but none in humans. Meanwhile for the immigration event scenario, Sect. 3.2, we consider the immigration of infected cattle with infected adult ticks during cultural events. We first explore the effects of different co-feeding proportions in Sect. 3.1.1 and then select a value to use to compare results for both baseline, Sect. 3.1.2, and immigration event scenarios, Sect. 3.2.

### Baseline Scenarios

#### Varying Co-Feeding

For simplicity in simulations and analyses, we take all co-feeding transmission coefficients to be the same, $\eta _{NL}= \eta _{LN}= \eta _{AA}= \eta$. To investigate the effects of co-feeding on disease transmission, we ran simulations for 5 years and varied η from 0 to 0.25, meaning 0% successful co-feeding transmission to 25% successful co-feeding transmission. Our simulations were baseline scenarios, in which the disease was present in the populations initially, except for humans. A summary of the effects of different co-feeding transmission coefficients on human outcomes is summarized in Table 4. We emphasize the effects on human transmission as humans are the only symptomatic hosts, and in Uganda, one confirmed human case constitutes a national response (Kizito et al. 2018; Balinandi et al. 2021). A summary of the effects of varying η for all hosts and tick classes is included in Table 5 in the Appendix. As η increases, human infections acquired from ticks rise much more rapidly than infections from cattle. When η increased from 0 to 0.25 transmission, there were more infected ticks in all life stages, more human infections and deaths, and higher peak seroprevalences at slightly earlier times. Peak human seroprevalence increased by 21.5% (from 9.4% to 11.42%), and deaths increased by 23.8% (from about 21 to 26). Among the non-human hosts, seroprevalence in cattle rose from 71% to 77.45%, and small mammal prevalence from 59% to 65.84%.

Larval prevalence increased from 1.1% to 1.49%, nymphal prevalence from 3.9% to 4.77%, and adult prevalence from 7.3% to 9.92%. The adult tick infected class is the most sensitive to η, which is of importance because they transmit the disease to humans. Human infections by ticks had an increase of 35.0%, from about 555 to 749 infections, while infections by cattle increased about 2.2%, from about 497 to about 508.

The timing of the peak seroprevalences and prevalences all occurred in the last year of the simulation, year 5, and shifted forward as η was increased. The shift was more subtle for humans, small mammals, larvae, and nymphs, only coming forward one to two days. For cattle the peak seroprevalence went from June 23 with no co-feeding transmission to June 19 with 0.25 successful co-feeding transmission. Adult tick peak prevalence showed the most dramatic change, shifting earlier almost two weeks, from August 13 to August 1. As the co-feeding transmission increased, and the prevalence in the adult ticks shifted earlier, the disease transmission cycle was accelerated, and the peak seroprevalence in humans and cattle moved forward. For subsequent simulations, we fixed the co-feeding transmission to be 0.05, which is consistent with co-feeding coefficients in Matser et al. (2009). We then considered the baseline simulation for 5 years with a fixed η=0.05.

**Table 4:** Summary of human infections and seroprevalence outcomes for different co-feeding transmission coefficients (η)

η	Infections by ticks	Infections by cattle	Deaths	Max. seroprevalence
0.00	555	497	21	9.44%
0.05	596	500	22	9.88%
0.10	639	503	23	10.39%
0.25	750	509	26	11.42%

#### Fixed Co-Feeding

**Fig. 4:**
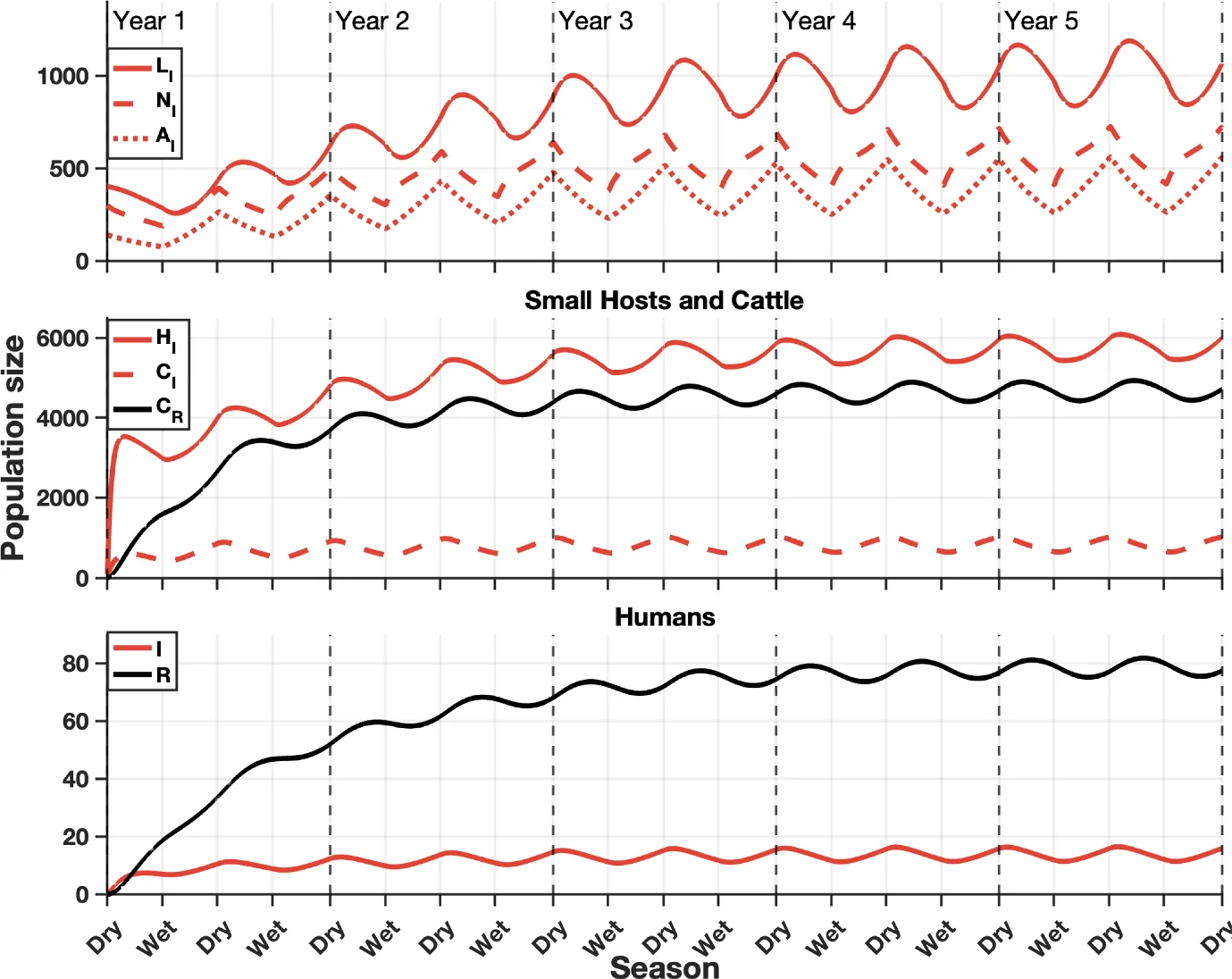
Time series of infection dynamics of the baseline scenario simulation by classes. The top panel displays the number of infected for all three life stages of ticks: larvae (solid line), nymphs (dashed line), adults (dotted line). The middle panel depicts the number of infected small hosts and infected and recovered cattle. Infected small hosts are displayed with a solid red line. Number of infected cattle are displayed with a dotted red line, and recovered cattle with a solid black line. The bottom panel shows the number of infected humans with a solid red line, and the number of recovered humans with a solid black line. The horizontal axis depicts the five year time frame by rainfall seasonality, with each tick representing the beginning of a wet or dry season. Dashed vertical lines highlight the transition between years, and years are labeled in the top panel (Color figure online)

**Fig. 5:**
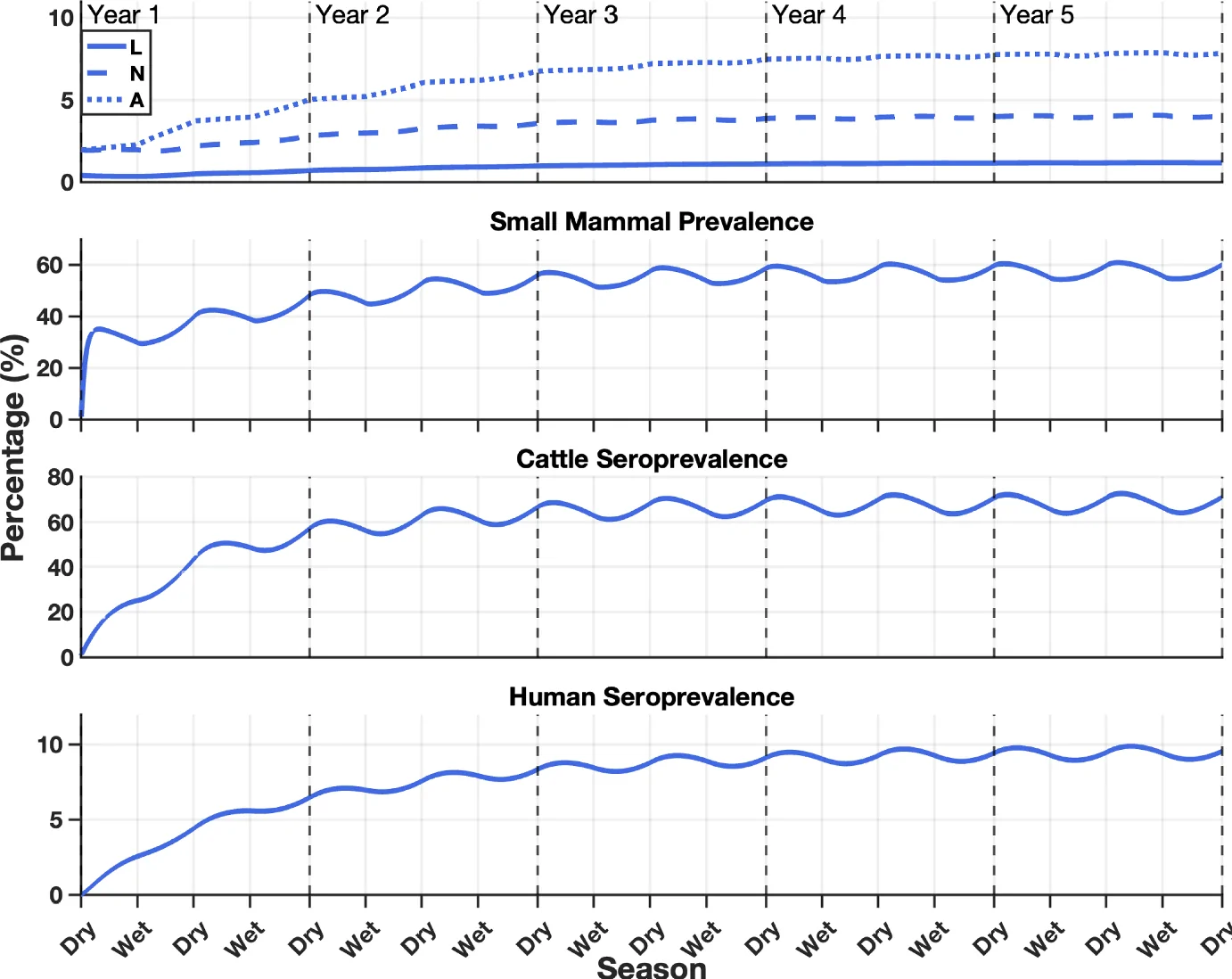
Seroprevalence and prevalence rates of the baseline scenario simulation are depicted with blue lines in four panels for different classes. The top panel displays the prevalence of infected for all three life stages of ticks: larvae (solid line), nymphs (dashed line), adults (dotted line). The second panel from the top displays the prevalence of small mammal hosts; the third panel from the top displays seroprevalence (sum of infected and recovered divided by the total) of cattle; the bottom panel displays the seroprevalence of humans. Dashed vertical lines highlight the transition between years, and years are labeled in the top panel (Color figure online)

For the case in which η is fixed at 0.05, we considered five-year baseline disease dynamics, in which CCHF was already circulating at low levels across multiple host and vector species. We assumed low levels of disease were present in all classes except humans, and assumed that no disease was initially present in the human population. Therefore, the model was initialized with only a small proportion of ticks, cattle, and small mammals infected, while the majority of each population remained susceptible. Humans were assumed to begin fully susceptible with no prior immunity. The following results are for a simulation of five years with the parameter choices in Tables 2 and 3 (Figs. 4 and 5).

Infection persisted in the system throughout the five-year simulation. The seasonal fluctuations in the tick population produced similar seasonal dynamics in host infection as seen in Figs. 4 and 5. Over the five-year simulation, transmission to humans was continuous, with a total of about 1,096 new infections. Approximately 596 infections were acquired via tick bites and about 500 through contact with infected cattle, resulting in approximately 22 human deaths and a case fatality rate (number of deaths / total number of infections) of 2.01%. By the end of the simulation, human seroprevalence stabilized near 10%, while seroprevalence and prevalence among cattle and small mammals reached about 71% and 60%, respectively. Tick infection prevalence was around 1% in larvae for the entire simulation. For nymphs and adults, prevalence increased steadily throughout the entire simulation, and ended around 4% for nymphs and 8% for adults. The timing of maximum prevalence also highlighted seasonal and interspecies differences. All hosts peaked in the mid-summer of year 5 (June–July). Small mammals peaked first in June, then cattle later in June, then humans in early July. Nymphs and adult ticks peaked in the late summer, nymphs in August of year 5, and adults in September of year 5. This indicated that the disease spread quickly to the small mammal population, while taking more time to cycle through to the later life stages of the ticks, which in turn cycled through cattle and humans.

We also investigated which transmission values could lead to less than one human death in a single year. We started with the base transmission values from ticks and cattle, $\beta _1$ and $\beta _2$ respectively, and multiplied these values by constants between 0 and 1. We found that when the transmission values were multiplied by.25, there was less than one human death in a year: that is to say, reducing the transmission values by 75% led to not a single human death in one year. When either cattle or tick transmission was held at its baseline value (Table 2), no reduction in the other transmissions was sufficient to reduce human deaths to less than one per year. Even setting one transmission value to zero while keeping the other at the baseline value (Table 2) still resulted in between one and two human deaths in one year.

### Immigration Event Scenarios

Next, we considered immigration event scenarios related to significant cultural events, as reports have indicated an increased transmission of CCHF due to increased slaughtering of livestock (Stone 2025). In 2016, twenty deaths from CCHF were attributed to an immigration event during Eid-Al Adha (Mallhi et al. 2016). To simulate these immigration event dynamics, we fixed two immigration events per year: one on December 25 corresponding to Christmas, and one on June 6 corresponding to the Eid-Al Adha festival, an annual cultural gathering. Eid-Al Adha occurs at different dates from May to July depending on the year, and we used June 6, as Eid-Al Adha was observed on this day in 2025. The following simulation was for 5 years, with the two holiday immigration events described above for each year. To aid comparing the two scenarios, we used the same initial conditions as our baseline scenario simulations, and for a finite number of times introduced cattle and adult ticks with high proportions of infecteds to the population. To illustrate the importing of cattle for slaughter and consumption during a holiday event, we introduced 250 cattle and 250 adult ticks, each with 200 susceptible and 50 infected. For a population of 1000 humans, this would correspond to most of the population engaging in the holiday.

**Fig. 6:**
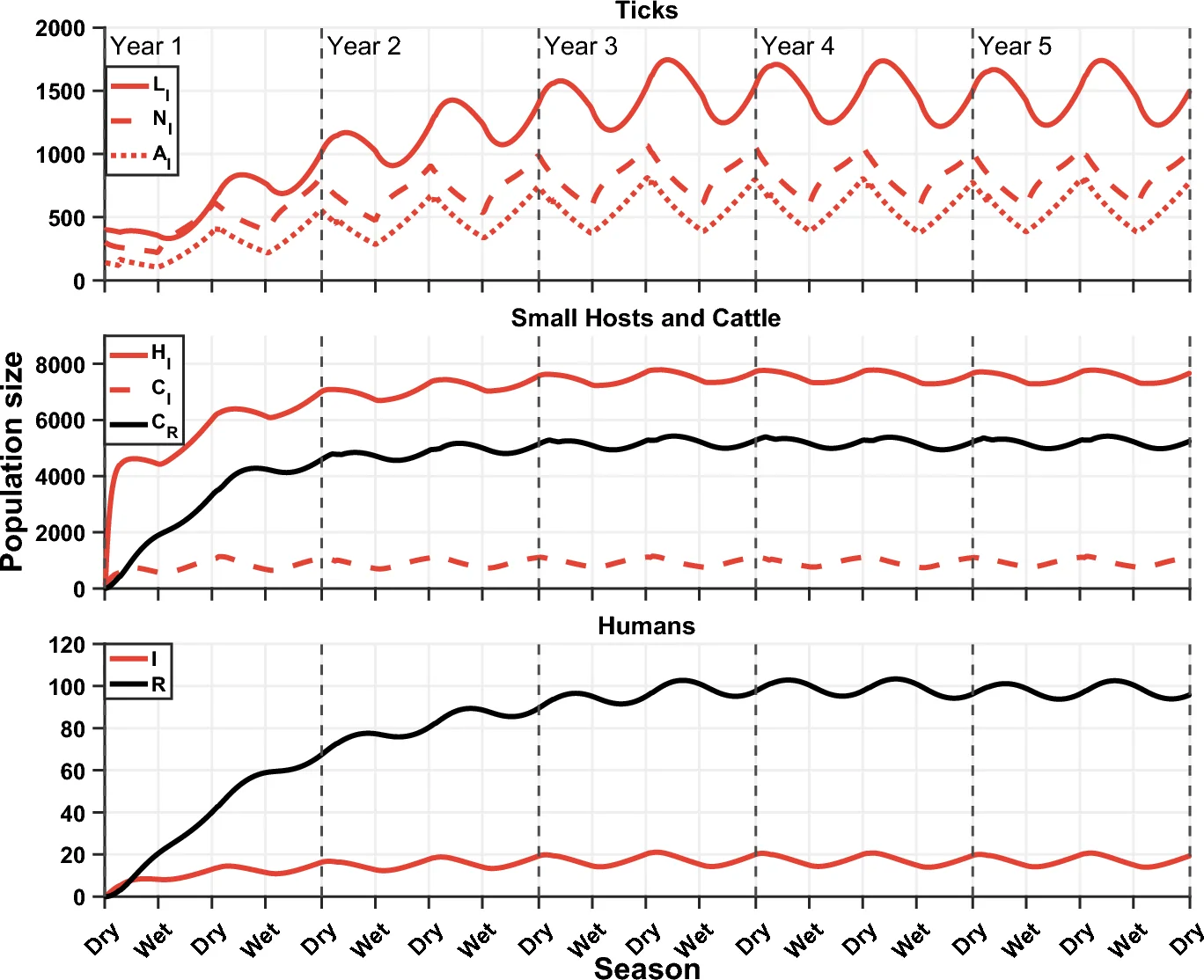
Time series of infection dynamics of the immigration event simulation by classes. The top panel displays the number of infected for all three life stages of ticks: larvae (solid line), nymphs (dashed line), adults (dotted line). The middle panel depicts the number of infected small hosts and infected and recovered cattle. Infected small hosts are displayed with a solid red line. Number of infected cattle are displayed with a dotted red line, and recovered cattle with a solid black line. The bottom panel shows the number of infected humans with a solid red line, and the number of recovered humans with a solid black line. The horizontal axis depicts the five year time frame by rainfall seasonality, with each tick representing the beginning of a wet or dry season. Dashed vertical lines highlight the transition between years, and years are labeled in the top panel (Color figure online)

**Fig. 7:**
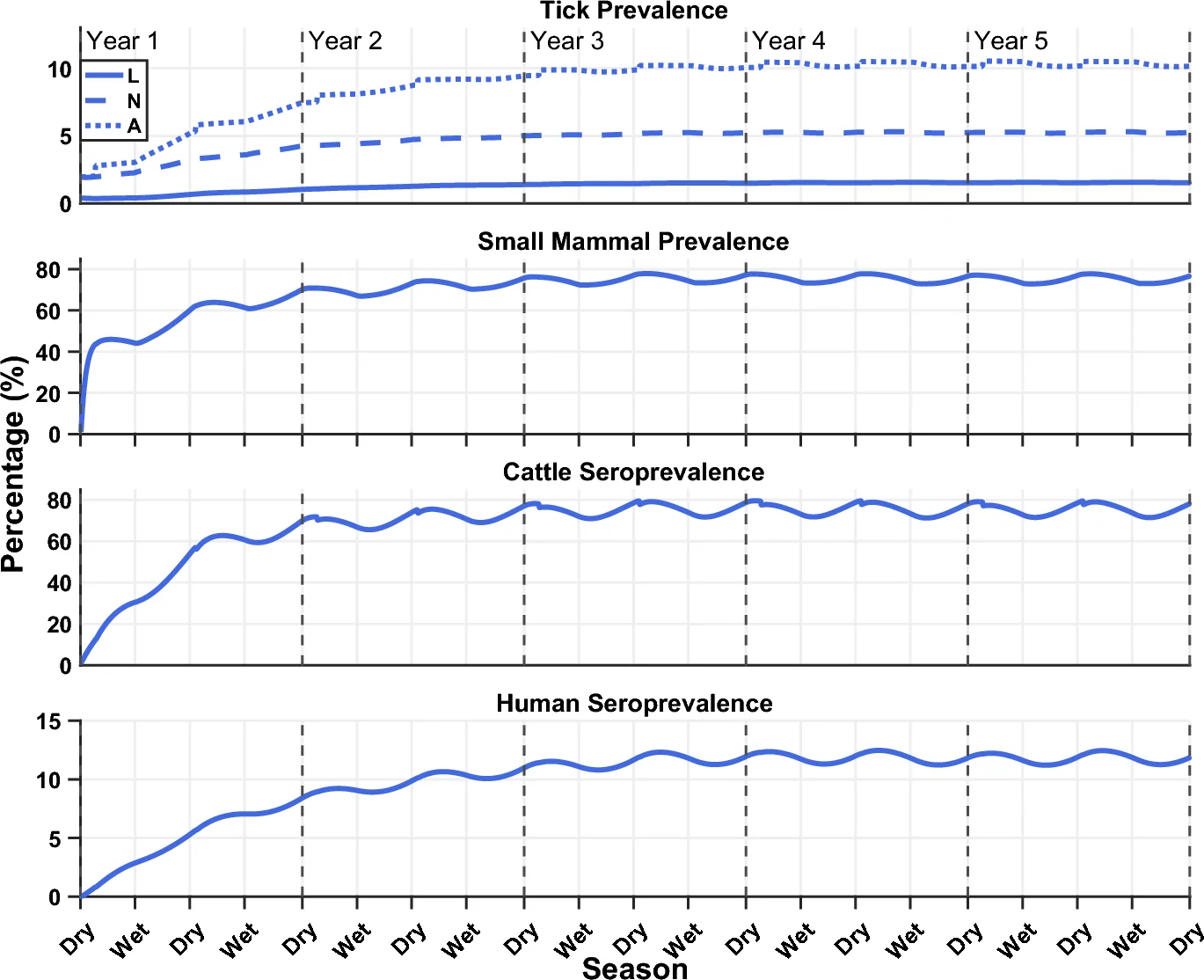
Seroprevalence and prevalence rates from the immigration event simulation are depicted with blue lines in four panels for different classes. The top panel displays the prevalence of infected for all three life stages of ticks: larvae (solid line), nymphs (dashed line), adults (dotted line). The second panel from the top displays the prevalence of small mammal hosts; the third panel from the top displays seroprevalence (sum of infected and recovered divided by the total) of cattle; the bottom panel displays the seroprevalence of humans. Dashed vertical lines highlight the transition between years, and years are labeled in the top panel (Color figure online)

Two immigration events per year produced persistent biannual epidemic waves across ticks, livestock, small mammals, and humans, rather than the monotonic approach to equilibrium seen in the baseline scenario runs (Figs. 6 and 7). There were about 1,392 total human infections over the 5-year period, about 878 from ticks and 514 from cattle, resulting in about 28 human deaths. Thus, there was a case fatality rate of 2.01%. The case fatality rate remained the same as in the baseline scenario case and the number of infections by cattle was similar; however, the number of infections by ticks increased by almost 300 cases. Compared to the baseline scenario simulation, humans had a higher seroprevalence that was reached more rapidly. There were fluctuations in the seroprevalence between about 11.25% and 12.25%, with maximum seroprevalence of 12.48% in July of Year 5. Because immigration events occurred twice per year, human peaks recurred at predictable intervals, about every 180 days. The quicker, higher-amplitude peaks corresponded to immigration event times; between peaks seroprevalence dropped to about the peak prevalence in the baseline scenario. Cattle sustained high seroprevalence with maximum 79.59% in December of year 4. The seroprevelance peaks were biannual to around 77% and then dropped to around 71% between immigration events. Small mammals also had high prevalence, with the maximum of 77.88%. The prevalence consistently dropped to 72% between immigration events and grew to about 77% biannually.

Larval prevalence stayed around 1.5% the entire simulation, with maximum 1.56% in September of year 5. Nymphal prevalence peaked at 5.3% in August of year 5. Adult ticks, which feed on cattle, attained the highest vector prevalence, with a maximum of 10.53% at the end of the simulation in December of year 5. These staggered peaks illustrate the progression of infection through the tick life cycle, ultimately reinforcing the biannual immigration event cycles observed in humans and livestock.

The baseline and immigration event scenarios differed most sharply in the magnitude and timing of human infection: under identical transmission parameters, the baseline produced a gradual long-term buildup, while immigration events drove sustained, recurrent surges. In the baseline case, infection persisted at low levels across all host and vector species, with prevalence patterns of tick abundance shaped by rainfall seasonality. Humans experienced about 1,096 infections over five years, with 569 acquired from ticks and 500 from cattle, leading to approximately 22 deaths and a case fatality rate of 2.01%. Human seroprevalence rose gradually and approached 10% by year five. Cattle and small mammals followed a similar increasing pattern to humans, with seroprevalence reaching 71% and 60%, respectively. Tick prevalence remained low in larvae and nymphs but increased steadily in adults, reaching about 8% by the end of the simulation. Importantly, the baseline system showed multiple seasonal peaks per year with a consistently increasing seasonal peaks. This reflects a gradual buildup of infection across hosts and vectors as the pathogen passes through successive tick life stages and maintains circulation in the hosts.

In immigration event simulation scenarios, the dynamics shifted dramatically due to periodic introductions of infected cattle and adult ticks. With immigration events fixed to occur twice yearly, the system exhibited sharp biannual epidemic waves that quickly moved infection towards maximum levels. Humans experienced about 1,392 infections over five years, with 878 tick-borne and 514 cattle-borne cases, leading to 28 deaths and the same case fatality rate of 2.01%. Unlike the gradual increase observed in the baseline case, human seroprevalence rose to near maximum early in the simulation and then oscillated between about 11.25% and 12.25% throughout. Cattle and small mammals likewise reached high seroprevalence quickly, with cattle peaking near 80% and small mammals near 78%, and then fluctuated between high values rather than continuing to climb. Small mammals saw the most dramatic increase in prevalence from baseline to immigration event scenario, with the prevalence increasing over 25% (from about 60% to 78%). Tick prevalence, particularly in adults, also synchronized tightly with immigration event times, reinforcing the biannual rhythm of epidemic surges.

Together, the baseline and immigration event simulations reveal distinct long-term patterns in CCHF transmission. Baseline transmission produced slow, seasonal increases in infection across hosts and vectors, with gradually rising seroprevalence in cattle, small mammals, and humans. In contrast, the introduction of biannual immigration events rapidly drove the system to high prevalence early on, after which sharp, predictable epidemic waves occurred biannually. This difference in system behavior has important implications: while baseline scenarios represent a persistent background risk, immigration event scenarios produce larger and more frequent surges in human infection, with the added risk of sudden increases in transmission coinciding with livestock handling during culturally significant events. Although transmission parameters were identical across scenarios, immigration events resulted in substantially more human infections from ticks than from cattle, emphasizing the importance of interventions timed around high-risk slaughtering events and the value of targeting adult tick populations. The unexpectedly large increases in small mammal prevalence under immigration event conditions, despite immigration events introducing infection only through adult ticks and cattle, underscore the tight coupling across hosts and the amplifying role of the earlier tick life stages.

## Discussion and Conclusions

Our model of CCHF includes all three life stages of ticks that feed, as well as human, cattle, and small mammal hosts. Co-feeding transmission of the virus is also included as a key pathway of transmission among ticks. We adapted our model to the Cattle Corridor in Uganda by considering reasonable host class sizes, rainfall seasonality in cycling tick populations, and plausible immigration events. By explicitly including details like co-feeding, seasonality, and immigration events, our simulations revealed dynamics that simpler models have not captured.

Our simulations presented two qualitatively distinct dynamical regimes: a seasonally governed baseline state and an immigration event-driven oscillatory state. In baseline settings, infection accumulates slowly across hosts and vectors, producing multi-peaked seasonal patterns and a steadily rising trend. Periodic introductions of infected cattle and adult ticks; however, rapidly push the system into persistent high-level oscillations that generate larger and more frequent human infection peaks. These results demonstrate how ecological influences, particularly introductions of infected adult ticks through mass movement of livestock, can shift CCHF transmission from low-level persistence to recurrent epidemic surges.

Previous CCHF models primarily focused on adult ticks and their hosts, yet our results show that earlier life stages of ticks play an important role in driving immigration event intensity, especially through transmission among small mammals. Co-feeding, in particular, is a critical transmission pathway that amplifies infection in ticks and can accelerate human infections. Similarly, seasonality in tick populations and host interactions created strong oscillations that shaped both baseline and immigration event scenarios. Immigration event simulations further revealed how periodic introductions of infected cattle and adult ticks shifted the system from gradual growth to quick peaks of infecteds. Taken together, these features demonstrate that models which ignore these ecological details risk underestimating both the persistence and the epidemic potential of CCHF.

Our exploration of co-feeding transmission demonstrates that even modest increases in the co-feeding transmission can noticeably and consistently amplify disease transmission dynamics across hosts and ticks, and its strongest effects appear in the tick-borne route of human infection. As the co-feeding transmission increased, the model shifted a growing share of human infections toward tick bites rather than cattle exposure. This pattern aligns with field observations: most confirmed CCHF cases documented by the WHO report a recent tick bite (WHO 2025), which emphasizes that direct tick-to-human transmission is typically the dominant pathway. In our model, enhanced co-feeding increases infection within the tick population, especially in the earlier life stages, and this within-vector amplification ultimately propagates outward to hosts. The resulting increases in human infections, deaths, and long-term seroprevalence illustrate that co-feeding is not merely a secondary mechanism but a process capable of strengthening viral circulation and altering the balance of transmission routes in meaningful ways.

The effects of co-feeding further clarify how virus transmission among ticks shapes infection patterns across the system. The sharper increases in human infections from ticks compared to cattle suggest that co-feeding primarily intensifies within-tick transmission loops, which then propagate outwards to hosts. The more modest increase in human infections from cattle can be explained by our ecological framework: cattle infect humans only after first acquiring infection from an adult tick, creating a more delayed or subtle pathway. As co-feeding increases the proportion of infected larvae and nymphs, more of these immature ticks mature into infected adults which almost immediately affects humans through direct feeding. In contrast, cattle-mediated transmission requires two steps (adult tick to cattle, then cattle to human), making its response to co-feeding less pronounced.

Our work extends co-feeding transmission modeling by examining a more complex ecological system that explicitly includes the earlier tick life stages and their hosts, small mammals. This provides new insights into how co-feeding can not only influence human transmission, but also transmission among other hosts and ticks. Our results highlight the importance of incorporating co-feeding into mathematical models of CCHF. Even subtle differences in the co-feeding transmission produced measurable changes across hosts and ticks, altered timing of seasonal peaks, and shifted the primary source of human infections to tick transmission. By including additional tick life stages in our co-feeding transmission dynamics, our results highlight how co-feeding strengthens viral circulation within ticks, and this within-tick transmission propagates to higher host infectivity.

Beyond co-feeding, seasonal variation in tick ecology also exerts a major influence on CCHF transmission. Vector seasonality is central to the timing and amplitude of prevalence and seroprevalence peaks in all hosts. In our model, we explicitly vary tick biting rates, tick mortality, and larval influx by rainfall (wet-dry) seasonal cycles. This produces the multi-peaked annual structure observed in the baseline runs and defines periods of greatest vulnerability during wet seasons, when tick activity and recruitment are highest. Seasonal fluctuations in tick populations have been studied extensively, including for *Hyalomma* ticks (Offei Addo et al. 2024; Estrada-Peña 2023). Our results emphasize the necessity to include seasonality in models of CCHF, as the seasonal fluctuations in tick populations strongly influence disease dynamics.

Comparing our prevalence and seroprevalence rates from our baseline and immigration event scenarios to reported rates of CCHF across the Cattle Corridor reveals several important points about the biological relevance of our model and calls for future data collection. Our results were consistent with some studies of CCHF in the Cattle Corridor, which found human seroprevalence around 10% and cattle seroprevalence within the range of our simulations (Lule et al. 2022). Other studies in the region however, reported substantially higher seroprevalence levels; for example, human seroprevalence approaching 30% and cattle seroprevalence exceeding 90% (Atim et al. 2022, 2023; Balinandi et al. 2021). These discrepancies likely reflect differences in sampling contexts, as higher values often come from testing triggered by a confirmed human case or targeted abattoir sampling. Reported case fatality rates also vary widely: while official surveillance in Uganda identified 14 confirmed cases and 5 deaths (36% case fatality) over a six-month period (Mirembe et al. 2021), such reports primarily capture severe cases of CCHF. Because approximately 80–90% of CCHF infections are believed to be subclinical (WHO 2025; Bodur et al. 2012), and only a small fraction of health facilities conduct viral hemorrhagic fever surveillance (Muleme et al. 2017), confirmed cases almost certainly underestimate true incidence, and inflation of apparent case fatality ratios is expected. Together, these comparisons suggest that our modeled seroprevalence values fall within the plausible epidemiological range while also highlighting the need for more systematic testing of humans, livestock, and ticks to better calibrate and validate future models.

Our study was limited by the availability and quality of data on key transmission and ecological parameters. We may also consider different parameter choices for co-feeding at different tick life stages; for example, increased co-feeding between larvae and nymphs compared to adults due to their higher abundance on small mammals could be considered. The uncertainties in our assumptions and parameter values underscore the urgent need for more robust empirical data to inform future models. Specifically, we encourage further investigation of co-feeding dynamics, as realistic co-feeding parameters are essential for capturing the true impact of CCHF. Information collected for CCHF across rainfall seasons, along with testing of small mammal hosts and immature ticks will be important for refining estimates of transmission and improving model accuracy.

From a public health perspective, two immediate implications emerge from this work. First, interventions that reduce tick abundance or tick-host contact would be favorable to disease control, especially measures targeted at adult ticks that feed on cattle and humans. When tick and cattle transmission values are reduced by 75%, our model predicts less than one human death from CCHF in a single year. Secondly, interventions timed around major slaughtering events like religious holidays can reduce surges in human infections. Preventative practices for CCHF motivated by immigration events during Eid-Al Adha have included restricting cattle movement, spraying cattle with pesticides, and wearing protective clothing to prevent tick bites during rendering (Leblebicioglu et al. 2015). Taken together, these implications highlight the value of mechanistic modeling for identifying intervention points to reduce human risk.

Despite data limitations, the modeling framework we present can be adapted to diverse ecological and geographic contexts. As demonstrated in our simulations, transmission dynamics can depend strongly on climate, tick feeding behavior, and host interactions, all of which vary across geographic regions. Our model provides a flexible platform that can be parameterized for other settings if modelers collaborate closely with empirical experts to capture local variation, particularly in seasonality and host–tick contact patterns. Such adaptations will allow the framework to be applied to regions with either lower livestock contact or more intense tick exposure. Ultimately, tailoring this model to local ecological realities will support more accurate forecasting of CCHF dynamics and strengthen the evidence base for targeted control measures.

## Appendix

**Table 5:** Maximum (Sero)prevalence and animal values when varying co-feeding transmission parameter (η)

Host	η	Max (Sero)prevalence	Number infected^a^	Total
Human	0	9.44%	93	980
	0.05	9.88%	97	980
	0.1	10.39%	102	979
	0.25	11.42%	112	977
Cattle	0	70.77%	5,719	8,081
	0.05	72.51%	5,860	8,081
	0.1	74.31%	6,005	8,081
	0.25	77.45%	6,259	8,081
Small mammals	0	59.18%	5,918	10,000
	0.05	60.85%	6,085	10,000
	0.1	62.68%	6,268	10,000
	0.25	65.84%	6,584	10,000
Larvae	0	1.09%	861	79,033
	0.05	1.17%	928	79,300
	0.1	1.26%	1,015	80,581
	0.25	1.49%	1,205	80,846
Nymphs	0	3.87%	454	11,740
	0.05	4.07%	478	11,743
	0.1	4.28%	510	11,922
	0.25	4.77%	566	11,873
Adults	0	7.29%	522	7,156
	0.05	7.85%	563	7,166
	0.1	8.42%	610	7,245
	0.25	9.92%	711	7,164

^a^Rounded to the nearest whole integer. For various values of co-feeding transmission, η, maximum (sero)prevalence and the values that were used to calculate it are recorded, number of infected and the total population. For humans and cattle, recovered individuals are included in the number of infected column, reflecting seroprevalence. The reported values correspond to the time at which maximum (sero)prevalence is achieved, which may differ among values of η and among species

See Table 5.
